# Does the correction angle affect hidden blood loss in HTO?

**DOI:** 10.1186/s13018-020-02071-0

**Published:** 2020-11-11

**Authors:** Zheng Li, Yannong Wang, Guanglei Cao, Shuai An, Mingli Feng, Liwei Wang, Xi Wang, Guangzhong Yang, Guanghan Gao, Shuai Wang, Xufeng Jiao, Lei Ding

**Affiliations:** 1 Department of Orthopaedics, Xuanwu Hospital Capital Medical University, Changchun Street, Xicheng District, Beijing, 100053 China; 2 Department of General Surgery, Beijing Guangwai Hospital of Xicheng District, Beijing, China; 3 Department of Orthopaedics, Beijing Chaoyang Emergency Medical Center, Beijing, China; 4 Department of Articular and Spinal Surgery, Huludao Center Hospital, Huludao, Liaoning China

**Keywords:** High tibial osteotomy, Hidden blood loss, Correction angle, Tranexamic acid, Tourniquet, Knee arthroscopic surgery

## Abstract

**Background:**

High tibial osteotomy (HTO) has a history of nearly 60 years and has been widely used in clinical practice. Biplanar open wedge high tibial osteotomy (BOWHTO), which evolved from HTO, is an important therapy for the knee osteoarthritis. In our previous research, we found that the decrease of hemoglobin levels after high tibial osteotomy ranges from between 17 to 41 g/L, but this is highly inconsistent with the intraoperative bleeding and postoperative drainage observed in clinical practice. The purpose of this study was to investigate the perioperative hidden blood loss (HBL) after biplanar open wedge high tibial osteotomy (BOWHTO), as well as to study the effect of the actual correction angle on blood loss.

**Methods:**

A retrospective analysis was performed on 21 patients who underwent BOWHTO for osteoarthritis of the knee due to proximal tibia deformity. Gross equation was used to calculate the perioperative total blood loss (TBL) and HBL. The actual correction angle was measured by postoperative anteroposterior radiograph. The correlation between HBL and correction angle was determined through correlation analysis.

**Results:**

The TBL was 823.5 ± 348.7 mL and the HBL was 601.6 ± 297.3 mL, total hemoglobin loss was 25.0 ± 10.7 g/L, and the mean HBL/patient’s blood volume (H/P) was 13.19 ± 5.56% for 21 patients. The correlation coefficient of correction angle and H/P is statistically significant (|*r*| = 0.678, *P* = 0.001).

**Conclusions:**

The actual total blood loss after BOWHTO was significantly higher than the observed, and the HBL was objective existent after BOWHTO. The proportion of H/P is positively correlated with the correction angle.

## Introduction

High tibial osteotomy (HTO) has a history of nearly 60 years [1] and has been widely used in clinical practice. Biplanar open wedge high tibial osteotomy (BOWHTO), which evolved from HTO, is an important therapy for the knee osteoarthritis. In 1987, Hernigou [2] reported a series of long-term follow-up studies of open wedge high tibial osteotomy for varus knee arthritis, with encouraging results. Compared with total knee arthroplasty (TKA) and unicompartmental knee arthroplasty (UKA) and other therapy, BOWHTO has the following advantages: simple technique, small incision injury, accurate correction of deformity, convenient adjustment of mechanical axis correction. It is particularly indicated for the treatment of unicompartmental degenerative arthritis in the young, active patient in whom implant arthroplasty may not be ideal [3–5]. Disadvantages include the need for a bone graft, increased risk of nonunion, and the possibility of loss of correction due to osteotomy collapse. But in recent paper [6], allograft combined with iliac crest aspirate provided low nonunion and delayed union rates. Furthermore, the present meta-analysis shows that BOWHTO has a satisfactory survival rate (> 90%) at 10 years follow-up [7]. However, it should be considered that a good survival rate allows no statement on the postoperative rapid recovery and shortened length of hospital stay.

It has been reported that the decrease of hemoglobin levels after osteotomy ranges between 17 to 41 g/L [8, 9], but this is highly inconsistent with the intraoperative bleeding and postoperative drainage observed in our clinical practice. It is uncertain whether this can be attributed to hidden blood loss (HBL). Since the concept of HBL was presented by Sehat et al. [10] in 2000, a number of studies have shown that HBL is an important component of TBL in orthopedic surgical procedures [10–13]. And it has been taken seriously in hip and knee replacement and even spine surgery. Thus, identifying patients at risk of pre-operative anemia is important because it can facilitate appropriate medical optimization.

The aims of this study were to investigate the HBL in BOWHTO and the relationship between the HBL and the correction angle.

## Materials and methods

### Patients

We collected the clinical data of 21 patients with knee osteoarthritis who underwent primary unilateral BOWHTO at the Department of Orthopaedics in Xuanwu Hospital of Capital Medical University from July 2019 to February 2020. Among them, eight were male and 13 were female with an average age of 50 ± 14 years. All the patients were free of hematological diseases that could severely affect blood coagulation.

Variables such as gender, age, height, weight, pre- and post-operative hematocrit (HCT), pre- and post-operative hemoglobin (HB), intra-operative blood loss, post-operative drain-blood volume, allogeneic blood volume, and reinfusion volume of drained blood were recorded.

### BOWHTO procedures

The operations were performed under intraspinal anesthesia with tourniquet pressure routinely set at 260 mmHg. The arthroscopy exploration was performed before the operation. The tourniquet was not used at the time of exploration; it was pressured before repairing meniscus, removing pleated or enlarged synovium, and repairing injury of articular cartilage surface when necessary. The tourniquet was released after operation and the joint cavity was probed again. The incision was closed after flushing the joint cavity.

BOWHTO was performed by a single surgeon. After the tourniquet was inflated, an anteromedial skin incision was made along the proximal tibia and carried through the sartorial fascia to the pes tendons. Sequentially, two Kirschner wires were inserted, anteriorly and posteriorly at the osteotomy level respectively, and were directed to the safe zone between the level of the tip of the fibular head and the circumference line of the fibula head under fluoroscopic guidance. The osteotomy was performed beneath the Kirschner wires using an oscillating saw until the osteotomy line extended within 10 mm medial to the lateral cortex of the tibia. Then the surgeon performed a biplanar osteotomy extending the osteotomy beneath the tibial tuberosity. Thin osteotomes were used to open the osteotomy and fixed with a Tomofix plate (Synthes, Oberdorf, Switzerland). The gap in the osteotomy site was filled with gelatin sponge when the gap was less than 2 cm. Tricortical autologous ilium graft filled the site; the gap was greater than or equal to 2 cm.

The tourniquet was released and hemostasis was completed before the incision was closed. The amount of intraoperative blood loss was recorded. Hemostatic and analgesic drugs containing tranexamic acid were locally injected together (tranexamic 0.5 g, parecoxib sodium 40 mg, ropivacaine hydrochloride 200 mg, oxycodone 10 mg, adrenaline 0.15 mg). A drainage tube was indwelled in the osteotomy gap for continuous negative pressure suction. The drainage tube was not routinely clamped.

After the operation, the wound was pressurized and bandaged, the bandaging was removed 24 h postoperatively and the drainage tube was removed 48 h postoperatively. The drainage volume and the total removal time were recorded. Functional exercises on the ankle and foot of the affected limb were initiated 2 h postoperatively. Six hours after the operation, weight-bearing exercises were performed, and an intermittent inflatable pressurization device was applied to lower extremities to assist in the prevention of deep venous thrombosis. A routine blood test was performed on the 1st, 3rd, and 5th day after surgery, and hemoglobin and HCT were recorded. If the patient still had signs of severe anemia within 1 week after the operation, a routine blood test was conducted again. Three days post-operation, vascular ultrasound was performed to assess signs of venous thrombosis of the lower limb. If lower limb vein thrombosis was found in the vascular ultrasound, then low molecular weight heparin (LMWH) anticoagulation therapy was given.

### Calculation of blood loss

The patient’s blood volume (PBV) was calculated by the formula:

$$ \mathrm{PBV}=k1\times \mathrm{height}\ \left({\mathrm{m}}^3\right)+k2\times \mathrm{weight}\ \left(\mathrm{kg}\right)+k3 $$

where *k*1 = 0.3669, *k*2 = 0.03219, *k*3 = 0.6041 for males; and *k*1 = 0.3561, *k*2 = 0.03308, *k*3 = 0.1833 for females. The total red blood cell volume was calculated as the hematocrit value multiplied by PBV. Any change in red cell volume can therefore be calculated from the change in hematocrit. The lowest value of HCT detected within 5 days after the operation was used as HCT_postop_ in the following formula to calculate total blood loss (TBL).

$$ \mathrm{TBL}=\mathrm{PBV}\times \left({\mathrm{HCT}}_{\mathrm{preop}}-{\mathrm{HCT}}_{\mathrm{postop}}\right)/{\mathrm{HCT}}_{\mathrm{preop}}+\mathrm{amount}\ \mathrm{of}\ \mathrm{blood}\ \mathrm{transfusion}; $$

$$ \mathrm{HBL}=\mathrm{TBL}-\mathrm{Postoperative}\ \mathrm{drainage}-\mathrm{Amount}\ \mathrm{of}\ \mathrm{intraoperative}\ \mathrm{blood}\ \mathrm{loss}; $$

Finally, the proportion of HBL to the patient’s blood volume was calculated as H/P = HBL/PBV

### Measuring correction angle

**Fig. 1:**
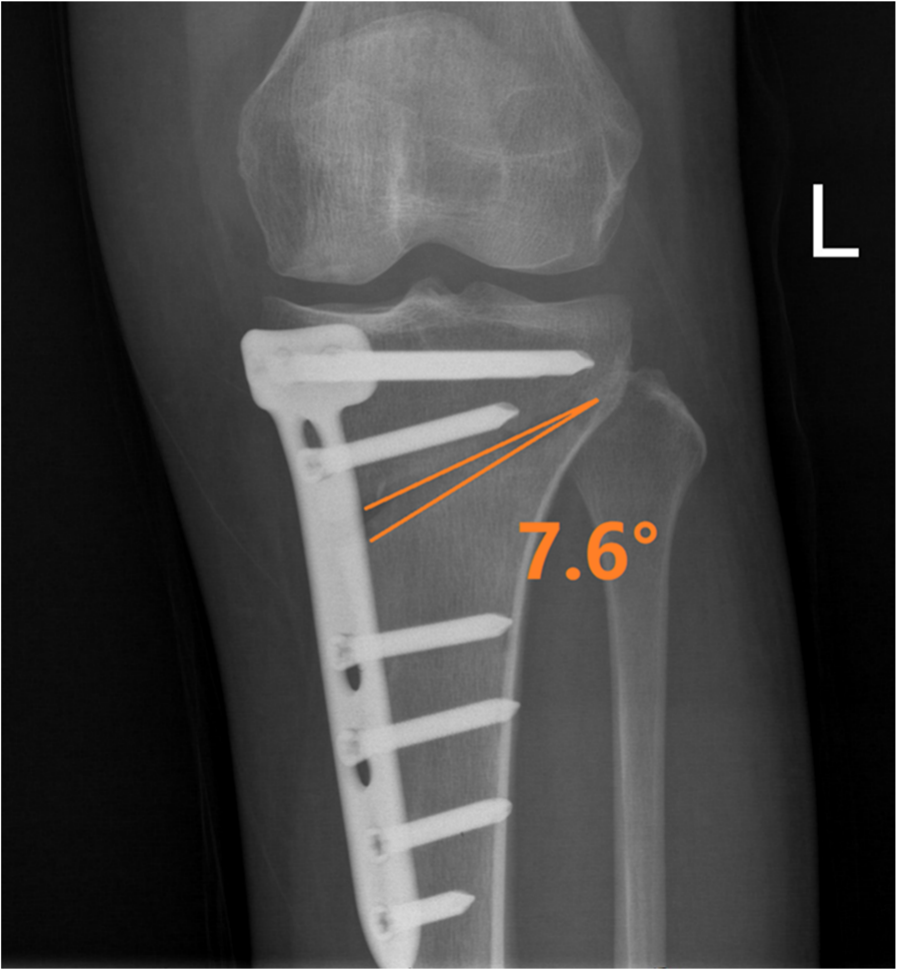
The correction angle was measured by standard orthotopic radiographs

The correction angle was measured by standard orthotopic radiographs (Fig. 1). The measurement was made by two observers, and the average was reported to reduce the observer error.

### Statistical analysis

Data analysis was performed using SPSS 23.0. Descriptive statistics were shown as mean ± standard deviation (SD) or number of cases and percentages when appropriate. Student’s *t* test for independent samples was used to compare TBL, HBL, and H/P between male and female patients. Correlational analyses were used to analyze the correlation between correction angle and TBL, correction angle and HBL, and correction angle and H/P. The correlation coefficient is expressed as |*r*|. The level of statistical significance was set at *P* < 0.05.

## Results

The mean TBL was 823.5 ± 348.7 mL, the mean HBL was 601.6 ± 297.3 mL, and the mean H/P was 13.19 ± 5.56% for 21 patients. The TBL 1086.96 ± 407.93 mL and the HBL 827.21 ± 296.71 mL in the male group were significantly higher than those of the female group, which were (694.00 ± 190.70 mL and 509.00 ± 173.11 mL), respectively (*P* = 0.07 for TBL, *P* = 0.06 for HBL). But the difference of H/P between the male group 15.75 ± 6.17% and the female group 11.63 ± 4.74 % was not statistically significant (*P* = 0.100) (Table 1).

**Table 1:** Difference of HBL, TBL, and H/P between male and female patients

	Male (*n* = 8)		Female (*n* = 13)		*P* value
	Mean	SD	Mean	SD	
TBL (mL)	1086.96	407.93	694.00	190.70	0.007
HBL (mL)	827.21	296.71	509.00	173.11	0.006
H/P (%)	15.75	6.17	11.63	4.74	0.100

*TBL* total blood loss, *HBL* hidden blood loss, *H/P* HBL/patient’s blood volume

**Fig. 2:**
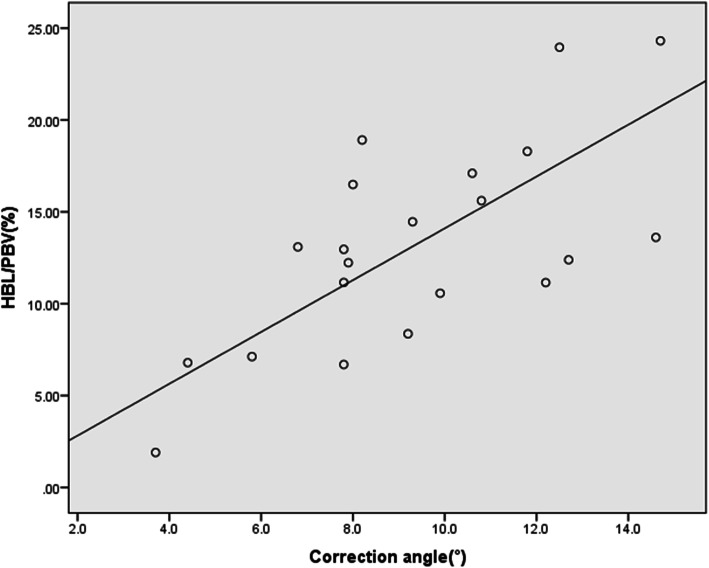
The correlation between correction angle and H/P

The correlation between correction angle and TBL, correction angle and HBL, and correction angle and H/P are shown in Table 2. The correlation coefficient of correction angle and H/P is statistically significant (|*r*| = 0.678, *P* = 0.001) (Fig. 2).

**Table 2:** The correlation between correction angle and TBL, HBL, or H/P

	|*r*|	*P* value
Correction angle—TBL	0.407	0.067
Correction angle—HBL	0.424	0.055
Correction angle—H/P	0.678	0.001

*TBL* total blood loss, *HBL* hidden blood loss, *H/P* HBL/patient’s blood volume

A total of 3 patients in this study were treated with vacuum sealing drainage because of the presence of wound fat liquid oozing due to poor wound healing. The wound healed after treatment. There were no cases of postoperative wound infection. Four cases of asymptomatic calf muscle vein thrombosis occurred after surgery. After the treatment of LMWH, no serious complications such as thrombus progression or pulmonary embolism were observed.

## Discussion

### Calculation of HBL

The Gross formula of calculating HBL is a linear model that accounts for circulating blood volume by using the perioperative mean hematocrit. It was first introduced by Ward [14] and later improved by Gross [15]. The calculation of perioperative blood loss by Gross formula is close to the reality. HCT is the most important reference index to calculate the HBL after BOWHTO. Preoperative HCT of patients is generally stable, but the changes of postoperative HCT vary greatly. At present, some studies suggested that it may be better to choose the 2-3 days postoperative HCT value because it is often the time point where the lowest value of postoperative HCT is observed. In this study, three-time points were selected to detect HCT: on the 1st, 3rd, and 5th day after the operation. This allowed for comparisons of dynamic blood volume changes, and then the lowest value of HCT detected among these measurements was selected for calculation.

### Comparison with other knee operations

As far as we know, this study is the first report to quantitatively analyze the HBL of BOWHTO. In previous studies regarding knee surgery, discussions of HBL mostly focused on UKA and TKA. Schwab et al. [16] and Zhang et al. [13] compared and studied the HBL of UKA and TKA. Schwab et al. argue that UKA has significantly less HBL than TKA. Zhang et al. compared 65 cases of UKA with 65 cases of TKA and also reported that HBL in the UKA group (375.25 ± 168.09 mL) was significantly less than that in the TKA group (898.81 ± 221.47 mL). However, as an important method for the treatment of knee osteoarthritis, HTO has not received a lot of attention to date. Suh et al. [8] reported a decrease of hemoglobin after high tibial osteotomy ranging from 17 to 41 g/L, but did not mention the problem of HBL. Kim et al. [17] found this phenomenon and believed that the discrepancy between blood loss and the degree of hemoglobin decline was caused by HBL, but did not conduct a quantitative analysis and elaboration on implicit blood loss. In our study, the TBL in BOWHTO was 823.5 ± 348.7 mL, the average HBL was 601.6 ± 297.3 mL, and the average hemoglobin decreased to 25.0 ± 10.7 g/L. This is similar to the results of Suh et al.’s paper. Compared with the study of Schwab et al., patients’ HBL after BOWHTO in our study was not only lower than that of TKA patients but also significantly higher than that of UKA. The HBL after BOWHTO is much higher than the dominant blood loss, which is closely related to the surgical method of osteotomy itself; factors such as intraoperative release of soft tissue, unsealed osteotomy sectioning, and routine application of tourniquet are the key factors that cause recessive blood loss [9, 18]. In clinical practice, we often find that patients can experience noticeable silt and redness at the distal end of the incision at 3-5 days after surgery that may even be combined with wound oozing, which is often caused by the accumulation of HBL under the skin and is a potential risk of wound infection. Therefore, HBL requires much greater attention in postoperative management and to minimize risks and ensure timely treatment of infection.

### Factors affecting HBL

In our study, the TBL and HBL of male patients were higher than those of female patients, which agree with results obtained in many other studies in orthopedic surgery. However, some authors [12] believe that the reason why TBL and the HBL are higher in male patients is that the circulating blood volume of most male patients was higher than that of female patients due to their height and weight. Thus, the TBL and the HBL are numerically different, but the ratio of total circulating blood should be similar as a result. In this study, H/P was also calculated and there was no significant difference in postoperative H/P between male and female patients. Therefore, it may be interpreted that the difference in HBL between male and female patients had no significant difference on systemic conditions. Individual differences of different heights and weights are common even in the population of patients of the same sex. Therefore, in all the studies of comparative HBL, H/P should be introduced for comparative analysis.

Furthermore, some authors [19] believe that age is a risk factor for increased blood loss after surgery because elderly patients may be more vulnerable to blood loss due to increased hypertension and the need to take aspirin orally, which both affect the coagulation and platelet function. However, this view is controversial. The data collected in this study were mainly derived from female patients. Meanwhile, due to the small sample size, patients of different age groups were not analyzed, which is another limitation of this study.

In a large number of studies [8, 17, 20, 21] on HBL in orthopedic lower limb surgery, tranexamic acid can effectively reduce total perioperative blood loss and hemoglobin levels in osteotomy. Studies have shown that tranexamic acid is an anti-fibrinolytic drug, which reversibly occupies the lysine binding site on the plasminogen molecule to prevent binding to fibrin. This in turn blocks fibrin degradation, thereby achieving hemostasis and reducing postoperative blood loss. In this study, tranexamic acid was used in all patients during the perioperative period, so it was not possible to analyze whether tranexamic acid reduces the HBL in BOWHTO. However, based on evidence from the medical literature, it is speculated that tranexamic acid is recommended to reduce postoperative blood loss in patients without contraindications.

### Correction angle

In this study, correlation analysis showed that the TBL, HBL, and H/P all had an upward trend with an increase of the correction angle. This may be because gelatin sponges or bone grafts are unable to fill the osteotomy wedge gap as the osteotomy correction angle increases. This result needs to be confirmed by a large cohort study in the future research to further guide clinicians to be conscious of patients with high correction angles of BOWHTO to avoid the occurrence of related complications.

There are several limitations in this study: it is a retrospective study, and its sample size is small, which may bias the relationship between gender, age, correction angle, and HBL. For tourniquet use, tranexamic acid use, and other potential influencing factors on HBL, it is not possible to conduct subgroup analyses, so the influence of these factors on BOWHTO’s implicit blood loss is not clear. Further research is needed to confirm these HBL trends.

## Conclusion

The actual blood loss after BOWHTO surgery is obviously higher than the visible blood loss (including postoperative drainage and intraoperative blood loss), and HBL is an objective measurement that may predict the risk of postoperative wound infection, which requires us to pay particular attention in clinical practice. At the same time, the increase of the correction angle may increase the amount of HBL. Hence, this should arouse the attention of the doctors in selecting treatment options to improve the prognosis of patients, such as the selection of bone grafting methods or blood management.

## Data Availability

The datasets used and/or analyzed during the current study are available from the corresponding author on reasonable request.
